# Innate Nectar Plant Attraction Is Primarily Visually‐Guided but Olfactory‐Stimulated in North American Monarch Butterflies

**DOI:** 10.1002/ece3.71533

**Published:** 2025-06-09

**Authors:** Darene A. E. Assadia, Delbert A. Green

**Affiliations:** ^1^ Department of Ecology and Evolutionary Biology University of Michigan—Ann Arbor Ann Arbor Michigan USA

**Keywords:** migration, monarch butterfly, multimodal signaling, sensory perception

## Abstract

In flower‐visiting insects, innate sensory preferences facilitate efficient foraging strategies in complex natural environments. Here we describe a nonforced choice assay to investigate innate attraction to a common nectar resource ( *Lantana camara* ) in naïve monarch butterflies ( *Danaus plexippus* ). We find that monarch butterflies have an innate attraction to *L. camara* in our assay. Visual cues are necessary and sufficient for sustained attraction at the tested range. However, olfactory cues increase the salience of visual cues for sustained attraction. The identities of the specific attractive visual or olfactory cues are not resolved. Altogether, this simple nonforced choice assay is suited to reveal quantitative differences in innate attraction in monarchs and, presumably, other insects.

## Graphical abstract

In flower‐visiting insects, innate preferences facilitate efficient foraging strategies in complex natural environments. We find that naïve monarch butterflies ( *Danaus plexippus* ) have an innate attraction to the nectar plant *Lantana camara* that primarily utilizes visual cues but is enhanced by olfactory cues.

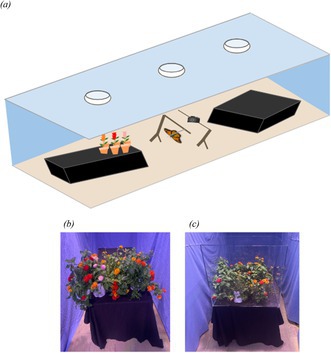

## 1 Introduction

Foraging requires complex, dynamic decision‐making that is guided by innate and learned behaviors. A major research goal is to understand how foragers will adapt to altered resource landscapes resulting from bottom‐up effects of climate change (Cuff et al. 2024). Learned behaviors play an important role in enabling behavioral flexibility for successfully foraging in changing environments, which can be especially important in foraging specialist species (Riffell et al. 2008). However, innate behaviors, or genetically controlled behaviors performed by naïve individuals with no prior experience with a stimulus, may have an important role to play as well because they can influence how new foraging behaviors are learned (Gumbert 2000), or they can evolve to enable flexible behavioral strategies that are broadly adaptive across different environments (Milet‐Pinheiro et al. 2016). For example, innate preference for specific colors by naïve animals was initially demonstrated over 100 years ago (Ilse and Vaidya 1956) and has subsequently been described in myriad species (e.g., Ilse and Vaidya 1956; Scherer and Kolb 1987a, 1987b).

Species are often cue‐specific in their sensory attraction, enabling adaptation to local foraging environments, particularly in specialist species (Rusman et al. 2024). Here we make the distinction between “attraction,” where individuals display seeking behavior in response to a stimulus, and “preferences,” where individuals show a stronger attraction among, or choose between, multiple putatively attractive stimuli. Flower‐visiting insects use a variety of cues from host plants—colors (Giurfa et al. 1995; Lunau and Maier 1995), odors (reviewed in Zjacic and Scholz 2022), shapes (Rausher 1978; Kunte 2007; Tiple et al. 2009; Dell'Aglio et al. 2016; Mukherjee and Hossain 2022; Mukherjee et al. 2024), patterns (Kelber 2002; Vaidya 1969), or even movements (Desai et al. 2024)—to facilitate foraging decisions. In some instances, specific focal cues predominate over others for foraging decisions, such as visual dominance in the red admiral butterfly (*Vanessa indica*; Ômura and Honda 2005) or skippers (family *Hesperiidae*) (Briggs et al. 2018; Tang et al. 2013). In many cases, however, multimodal cues are integrated (reviewed in Leonard and Masek 2014). Male hawkmoths (Raguso and Willis 2005), honeybees, and bumblebees (reviewed in Leonard and Masek 2014), for example, have evolved innate preferences for integrated visual and olfactory cues from relevant nectaring or pollinator host plants. Innate foraging preferences can be context‐specific, altered by multimodal cues (e.g., Vaidya 1969; Kuenzinger et al. 2019), spatial context (Kelber 1997), or internal state (Buehlmann and Graham 2022; reviewed in Kadow 2019). Innate foraging preferences for color can differ depending on visual context, such as in the pipevine swallowtail ( *Battus philenor* ; Briggs et al. 2018), or on olfactory environment, as in a swallowtail butterfly (*Papiliio xuthus*; Yoshida et al. 2015) and an obligate fruit‐feeding Satyrine butterfly (*Mycalesis maneus*; Balamurali et al. 2019).

Innate preferences are also important for foraging decisions in generalist species, although the task differs from that of specialist species. Generalists still must identify certain nectar resources among a varied landscape, as well as adjust choices as environments change. Honeybees ( *Apis mellifera* ) and bumblebees (*Bombus* spp.) have long been models for how innate preferences shape foraging behaviors and cognition more broadly (Leonard and Masek 2014). Generalist hoverfly species have been an important model for understanding how innate attraction (i.e., attraction of naïve individuals) changes across environments. The marmalade hoverfly (*Episyrphus balteatus*) and drone fly ( *Eristalis tenax* ) are attracted to “ubiquitous” multimodal cue combinations that can occur across different (hemiboreal, alpine, and tropical) environments, revealing a generalized resource identification strategy (Nordström et al. 2017). Less well understood is how innate preferences are utilized in species where foraging environments show strong temporal and/or spatial variation. In one example of this, Milet‐Pinheiro et al. (2016) found that the bivoltine bee *Andrena bicolor* , which switches between generalist (polylactic) and specialist (oligolectic) feeding strategies in different generations, shows similar reliance on visual and olfactory cues and does not adopt specific attraction behaviors across generations. There is a need to add to this knowledge gap by studying innate attraction in generalist forager species that experience highly temporally and spatially varying foraging environments.

Monarch butterflies ( *Danaus plexippus* ) present an interesting case for studying sensory perception, attraction, and preferences because of their unique migration and life history. Each autumn, millions of monarchs migrate up to 4000 km to reach specific overwintering sites in the Transvolcanic Belt of central Mexico (Urquhart and Urquhart 1978; Brower et al. 1977). Individuals spend winter in dense clusters, and in the following spring, they mate and remigrate northward, eventually repopulating their northern range over 2–4 partially overlapping generations (Malcolm et al. 1993). Thus, not only do distinct generations of monarchs experience different seasons, but also individual migratory butterflies live long enough to experience the distinctive foraging environments of autumn, winter, and spring. Adult monarch butterflies are generalist nectar feeders (Adamson et al. 2018; Brower et al. 2006). Different generations of monarchs forage in different environments and thus might be expected to have different capacities to perceive and respond to nectar plants. In late summer, monarchs are nonmigratory and exhibit station‐keeping behaviors where they usually remain within restricted ranges and forage locally (Zalucki and Kitching 1982, 1984; Grant et al. 2022). Autumn migrants, on the other hand, exhibit ranging behaviors, constantly encountering new foraging environments during their migration (Adamson et al. 2018; Grant et al. 2022).

Evidence of innate and learned behaviors that monarchs may employ when foraging is growing. Monarchs are attracted (presumably innately) to visual and olfactory cues from their milkweed host plants (Garlick 2007). Although visual and olfactory cues alone are sufficient for perception, attraction is maximized when both cues are available together (Garlick 2007). Monarch perceptual ability is likely dominated by vision at a fine scale, within 5 m of a resource (Garlick 2007; Fisher and Bradbury 2021). Field observations suggest that olfactory perceptual range is important at longer distances in natural settings (Fisher and Bradbury 2021). Monarchs have a strong innate preference for orange color, but this preference is context‐dependent (e.g., available color palette influences preference) and readily modified (Blackiston et al. 2011). They can learn nonpreferred colors equally as well as preferred colors and can readily learn to switch color associations with a reward (Blackiston et al. 2011). Male monarchs can learn visual and olfactory cues and store information in long‐term memory for at least 1 week, while only migratory females have this ability (Gegear 2021). What these investigations leave open, however, is how monarchs use sensory cues to identify nectar resources and how these cues may differ from other fitness‐related behaviors [e.g., oviposition (Garlick 2007)].

These studies represent a range of methods used to measure perception in monarchs and insects more broadly, including a range of field observations (Fisher and Bradbury 2021, 2022; Fisher et al. 2020, 2021), seminatural tests (Garlick 2007), and highly controlled laboratory studies (Blackiston et al. 2011; Gegear 2021). Seminatural tests that combine natural elements in a controlled environment (e.g., natural flowers versus artificial flowers in a laboratory study) are especially useful experimental paradigms because they can be expected to approximate behaviors under natural conditions yet readily allow assessment of the individual or combined effects of environmental factors on behavior (Opp and Prokopy 1986). Seminatural tests have been utilized less often to study perception in monarchs but nonetheless have provided valuable insights (Garlick 2007).

Here, we describe and test a seminatural nonforced choice assay to investigate “innate sensory perception” of a common nectar resource ( *Lantana camara* ) in monarch butterflies. We emphasize the distinction that individuals are born with innate sensory perception capabilities (i.e., genetically encoded morphological and physiological mechanisms to perceive and process sensory stimuli), but exposure to and experience with stimuli can change how sensory information is both perceived (by plastically altering sensory physiology (Gadenne et al. 2016)) and processed (by changing how sensory information is interpreted in the brain, e.g., Minoli et al. 2012; Anton et al. 2016; Jernigan et al. 2019). The nonforced choice nature of this assay enables us to study temporal behavioral dynamics while butterflies remain naïve to reward. *L. camara* is native to the southern United States (Taylor et al. 2012), yet also occurs in other parts of North America where they are planted as ornamentals. Monarchs have been reported to nectar on *Lantana* spp. across its North American migratory range (according to citizen science observations from iNaturalist). In this assay, butterflies were not allowed to land or receive a reward, so choices in this experiment represented innate behaviors and not associative learning behaviors. We assessed innate sensory perception by testing naïve monarchs (no foraging, nectar source exposure, feeding experience, or training). As an initial proof‐of‐principle of the assay, we conducted tests on individuals that were reared in an outdoor insectary in summer (nonmigratory) conditions. We tested the relative importance of composite visual and olfactory cues, and their integration, for close‐range nectar plant attraction.

## 2 Materials and Methods

### 2.1 Butterfly Collection and Husbandry

Adult monarchs of the parental generation were collected from various locations near Ann Arbor, MI, in June 2022, 2023, and 2024. No permits are currently required for collecting monarch butterflies in Michigan. Butterflies were hand‐fed daily an artificial nectar solution (Bird's Choice brand; 16.7 g of powder per 170 mL water) that includes a proprietary combination of glucose, fructose, calcium salt, halide salt, and amino acids. Rearing was conducted in an outdoor insectary that was isolated from flowering plants. Mating cages were established for egg collection on common milkweed, *Asclepias syriaca* . Caterpillars were reared on locally collected, bleach‐cleaned cuttings of *A. syriaca* . We monitored for infection by the protozoan parasite *Ophryocystis elektroscirrha* (OE) in all tested butterflies. All butterflies in these tests were uninfected by OE.

### 2.2 Flight Arena

**FIGURE 1:**
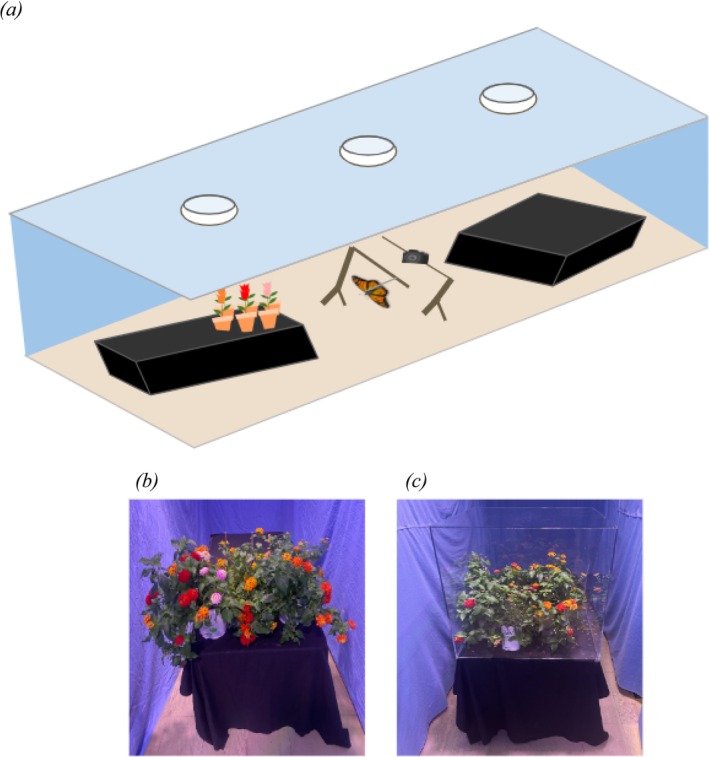
Visual displays of the behavioral arena. (a) This image shows an example of the interior arena containing whole, uncovered plants. (b) This image shows the combination treatment, with both visual and olfactory cues. (c) This image shows the visual‐only treatment, in which plants were covered with a UV‐transmitting plexiglass box that permits visual cues only.

The flight arena was set up in a light‐controlled indoor classroom (Figure 1a). The arena was lined with sky blue fabric in a rectangular pattern to limit visible distractions. Three full‐spectrum lights (LBW Grow Light, Full Spectrum, 150 W) were hung in the arena, one above the tethered monarch and one 2 m to the left and right of the monarch (above the respective treatments or controls). A monarch was tethered with thread to a tripod in the middle of the arena (Figure 1a). Treatment or control setups were placed on tables covered with black fabric to the left and right of the monarch (Figure 1b,c, treatment groups). Tables were moved to the respective distances of each trial (1 m or 3 m). Before each trial started, the space was aired out to eliminate olfactory cues from the previous trial.

Potted blooming *L. camara* plants (Proven Winners; Carleton, MI) were used as the nectar resource. *L. camara* specifically has been used as a nectar source for rearing and experimental monarchs in previous studies (James 1986; Robertson et al. 2015). Each trial used the following plants: one 2.5″ pot of the Luscious Pinkberry Blend, one 2.5″ pot of the Luscious Marmalade, one 4.5″ pot of the Luscious Royale Red Zone, and three 4.5″ pots of the Luscious Citrus Blend. One hundred and ten flower buds were counted before each trial. If there were more than 110, the excess flowering buds were removed. We note that *L. camara* does change flower color over the first few days upon first opening (Weiss 1997). Nevertheless, the plants used in this study were mature and at least 7 days beyond flower opening.

### 2.3 Flight Assay

Newly eclosed monarchs were kept in large pop‐up cages and separated by sex. All butterflies tested in the arena (*n* = 105) were starved for 48 h before testing. Most individuals (*n* = 56/105) were tested 48 h post‐eclosion. In cases where it was impossible to run all individuals within 1 day, untested individuals were hand‐fed artificial nectar daily, then returned to the holding cages. When ready for testing, individuals were starved for 48 h prior to the testing day. Throughout the study, individuals were tested up to 9 days post‐eclosion. Altogether, no butterflies had any exposure to or experience with any nectaring plant before the trials.

At the start of each trial, monarchs were placed in a neutral position (in the center of the arena and facing orthogonally to either treatment or control) within the arena for less than 5 s before being released. Individuals were tethered to a tripod in the center of the arena. The tether consisted of a white polyester thread (7 cm length) glued to a 2 cm^2^ bandage (Band‐Aid) piece attached to the thorax (Parlin et al. 2021) (Figure 1a). Monarch flights were recorded by an AKASO EK7000 camera placed overhead. Videos were recorded for 3 min. If the monarch exhibited hanging behavior, was entangled within the thread, or was otherwise stuck or stopped flying during the trial, it was placed back into the neutral starting position and encouraged to fly again. If the monarch still did not fly, it was removed from the arena, and after a 3‐min break, the trial was attempted once more. All individuals that flew directionally for more than 5 s were included within the dataset. Experiments were performed between August 3–13, 2022, July 28–31, 2023, and July 13–16, 2024.

### 2.4 Treatments

All butterflies were randomly assigned to a treatment and a distance. Butterflies were not tested again after a successful flight trial. Therefore, all groups consisted of a distinct set of individual butterflies, and there were no repeated measures. The experiment consisted of two treatments: Treatment 1 was a “combination” treatment (visual plus olfactory cues) consisting of whole, uncovered potted plants (Figure 1b). The control for this treatment was an empty table with no plants. Treatment 2 was a “visual‐only” treatment consisting of the potted plants (following the same criteria as the combination treatment) covered by a UV‐transmitting plexiglass box (Figure 1c). The control for treatment 2 was a UV‐transmitting plexiglass box with no plants inside. Placement of the treatment and control on either side of the room was randomized throughout the experiment using a random number generator. The combination treatment was tested at 1 m and 3 m distances, and the visual‐only group was tested only at 1 m.

### 2.5 Ethogram Construction and Analysis

Attraction behavior was assessed by summing directional flight over the length of the video. All videos were scored manually using a behavioral ethogram. Videos with less than 5 s of flight were removed from the analysis. Videos were scored for three primary behaviors observed by the authors of this study, as indicated in Table 1: directional flight, circling, and hanging. Total seconds of all behaviors were recorded and summed. The following measurements were calculated: directional flight toward plant (FP), directional flight toward control (FC), total directional flight (FP+FC), proportion of total directional flight in video ((FP+FC)/[video length]), proportion of directional flight toward plant (FP/(FP+FC)), proportion directional flight toward control FC/(FP+FC), total circling (C), total hanging (H), proportion circling in video (C/[video length]), and proportion hanging in video (H/[video length]). Note that (FP+FC+C+H) /[video length] = 1.

**TABLE 1:** Description of the behaviors that were observed and used to score videos.

Behavior	Description
Directional flight	Flight (active wingbeat) toward the treatment or control sides of the room (the video frame was bisected); direction of flight was determined by the head direction/body axis orientation
Circling	Flight in quick circles; circling was not incorporated into flight time
Hanging	No active wingbeat in video; hanging was not incorporated into flight time

### 2.6 Data Analysis and Statistics

All statistics were calculated in R (v. 4.1.2). All videos less than 3 min were removed from the data set, yielding 95 videos for analysis. Linear models were run in R (using *lm*) to initially investigate the dataset. The response variable was the proportion of time directed toward the plant, and treatment, sex, year, distance, age (i.e., days post‐eclosion of test) and treatment location (i.e., side of the room on which the treatment was placed, either the right or left of the monarch) were independent variables. Distance was found to be highly significant in this model (*p* = 5.92e‐05; Table 2). One specific age (7 days) was found to be significant as well (*p* = 0.0215). This group contained only two individuals (FP = 0.9545 and 0.6153). Given the small sample of this group and the one extreme value, we consider this instance of significance to be spurious and thus interpret these data to indicate that age is not a significant factor in this experiment. We proceeded to analyze the data using a new “group” variable, which combined all individuals tested in a particular treatment at a specific distance, yielding three groups: 1 m‐combination, 1 m‐visual, and 3 m‐combination. ANOVA was used to compare group means, followed by Tukey's Honestly Significant Difference (HSD) post hoc tests for pairwise group comparisons where significant differences were detected. A one‐sample *t*‐test was used to determine if each group significantly differed from random (proportion = 0.5).

**TABLE 2:** Linear model analyzing effects of year, sex, distance, treatment location, and age on the proportion of directional flight toward treatment.

	Estimate	Std. error	*t* value	Pr(>|t|)
(Intercept)	0.59333	0.06061	9.789	2.21e‐15***
Year: 2023	0.11521	0.08789	1.311	0.1936
Year: 2024	0.07052	0.07197	0.980	0.3301
Sex: f	−0.01678	0.03563	−0.471	0.6389
Distance: 3 m	−0.21963	0.05182	−4.239	5.92e‐05***
Treatment: visual	−0.06179	0.05194	−1.190	0.2376
Treatment location: down	0.01373	0.03973	0.346	0.7305
Post‐eclosion test day: 3	0.05975	0.09028	0.662	0.5100
Post‐eclosion test day: 4	0.11478	0.08545	1.343	0.1829
Post‐eclosion test day: 5	0.15492	0.08619	1.798	0.0760
Post‐eclosion test day: 6	0.15123	0.11337	1.334	0.1859
Post‐eclosion test day: 7	0.30985	0.13212	2.345	0.0215*
Post‐eclosion test day: 8	0.13334	0.08822	1.511	0.1346

* *p* ≤ 0.05.

*** *p* ≤ 0.001.

Additionally, we investigated if the propensity to fly toward the plant differed over the course of the 3‐min trial. Analyzing flight data temporally, even at coarse resolution, can reveal important behavioral changes that might otherwise go unnoticed (e.g., McKenzie‐Smith et al. 2025). To assess this, we first removed all hanging and circling data from the ethogram to yield only directional flight, then bisected each individual's directional flight into two equal halves. We then calculated the proportion of flight directed toward the plant for each half and took the difference of these values (FP/(FP+FC)^second‐half^−FP/(FP+FC)^first‐half^). This value ranges from −1 to 1 with an expectation of 0 if the proportion of flight toward the plant was equal across halves, positive if attraction to the plant increased over time, and negative if attraction to the plant decreased over time. A one‐sample *t*‐test was used to determine if each group significantly differed from proportion = 0.

## 3 Results

### 3.1 Innate Attraction to *L. camara*

**FIGURE 2:**
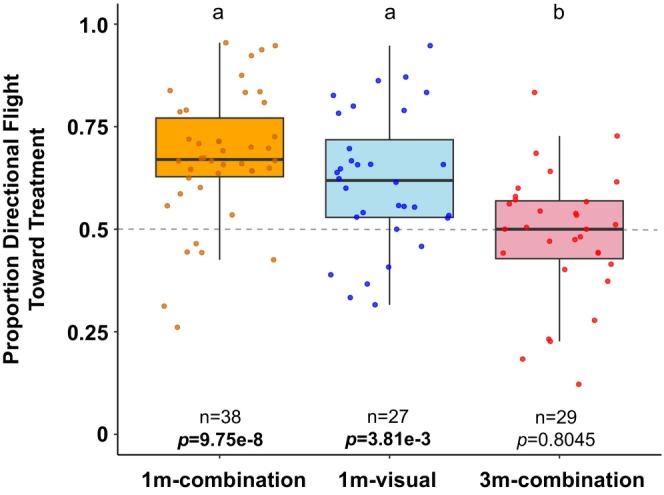
Proportion of directional flight toward the treatment. Each point represents an individual. A proportion of 0.5 indicates random choice/no preference. Plots indicate median (black horizontal bar) and 25th and 75th percentiles (lower and upper box bounds, respectively) of values.

**FIGURE 3:**
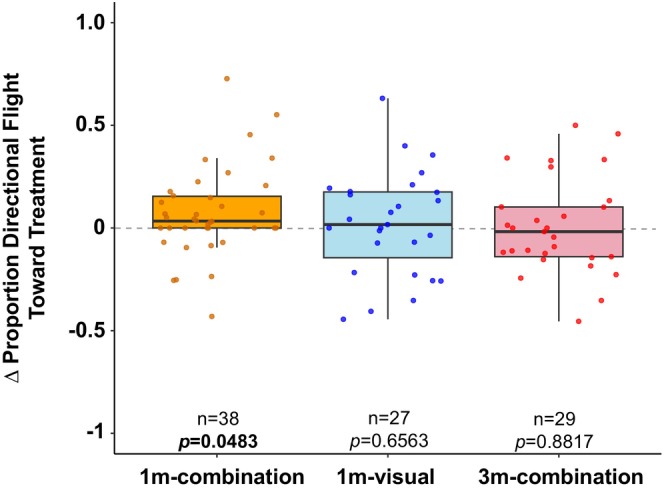
Proportion of directional flight toward the treatment in the second half of the video minus the first half of the video. Each point represents an individual. A value of 0 indicates that the proportion of flight toward the treatment did not change over time; a positive value indicates that attraction to the treatment increased over time; and a negative value indicates that attraction to the treatment decreased over time. Plots indicate median (black horizontal bar) and 25th and 75th percentiles (lower and upper box bounds, respectively) of values.

We first tested whether monarchs exhibited an attraction response to *L. camara* plants in our indoor arena. We assumed, as has been done before in similar assays (Brower et al. 1977), that an attractive stimulus should elicit *prolonged* investigation in the direction of the stimulus (Grant et al. 2022; Nathan et al. 2008). Monarchs showed prolonged flight in the direction of the plant versus control direction when the plant was located 1 m away (*t* = 6.60, df = 37, *p* = 9.75e‐8; Figure 2). The proportion of flight directed toward the uncovered plant in the 1 m‐combination group was higher in the second time bin (mean ± SEM across individuals: 0.7091 ± 0.0313) compared to the first time bin (mean ± SEM across individuals: 0.6369 ± 0.0339) (*t* = 2.04, df = 37, *p* = 0.0483; Figure 3), indicating increased attraction to the plant during a trial in this assay. The side of the room on which the plant was placed did not affect directional flight toward the plant (Table 2; *p* = 0.7305). Directional flight toward the plant did not differ between sexes (Table 2; *p* = 0.6389).

In order to test the limits of attraction behavior in this assay, we tested flight direction at a second distance farther from the treatment (3 m). Distance had a significant effect on flight toward the plant (*f* = 10.33, df = 2, *p* = 5.08e‐05; Figure 2). Monarchs lost their attraction to the plant when it was moved farther away (3 m) from tested individuals (*t* = 0.2500, df = 28, *p* = 0.8045; Figure 2). There was no difference in the proportion of flight directed toward the uncovered plant between the first half (mean ± SEM across individuals: 0.5089 ± 0.0328) and second half (mean ± SEM across individuals: 0.5154 ± 0.0380) of flight (*t* = 0.1501, df = 28, *p* = 0.8817; Figure 3) in the 3 m‐combination group.

**FIGURE 4:**
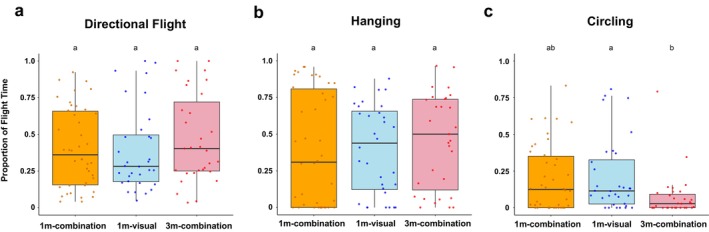
Proportion of (a) total directional flight, (b) hanging, and (c) circling in the trial per individual. Plots indicate median (black horizontal bar) and 25th and 75th percentiles (lower and upper box bounds, respectively) of values.

There were no significant differences across groups in the proportion of time spent flying directionally (*f* = 1.185, df = 2, *p* = 0.31; Figure 4a) or in the proportion of time spent hanging in the arena (*f* = 0.181, df = 2, *p* = 0.834; Figure 4b). The proportion of circling, however, significantly differed across groups (*f* = 4.172, df = 2, *p* = 0.0185). Butterflies showed the lowest proportion of circling in the 3 m‐combination treatment (Figure 4c), which was significantly lower than in the 1 m‐visual group (*p* = 0.0178; Figure 4c) and approaching significance compared to the 1 m‐combination group (*p* = 0.0929).

### 3.2 Visual Cues Are Sufficient for Innate Plant Attraction, but Made Stronger With Olfactory Cues

We tested a “visual‐only” stimulus by obscuring olfactory cues with a transparent box to cover the plant. A similar box was placed at the same distance (1 m) on the opposite side of the arena to control for potential reflection effects from the transparent box. Monarchs showed sustained attraction to the covered plant in the 1 m‐visual group (*t* = 3.18 df = 26, *p* = 0.0038; Figure 2), indicating the sufficiency of visual cues for attraction behavior. The proportion of flight toward the treatment in 1 m‐visual was slightly reduced compared to 1 m‐combination, but the difference was not significant (*p* = 0.1980; Figure 2). Proportion of flight directed toward the covered plant in the 1 m‐visual group did not significantly differ between the first time bin(mean ± SEM across individuals: 0.5935 ± 0.0436) and second time bin (mean ± SEM across individuals: 0.6155 ± 0.0376) of flight (*t* = 0.4501, df = 26, *p* = 0.6563).

## 4 Discussion

We show that naïve monarchs have an innate attraction to *L. camara* , a common nectar resource. Since all tested butterflies were naïve to any type of nectar plant at the time of testing and did not ever receive a reward in this experiment, attraction in this experiment represents innate behaviors and not associatively learned behaviors. We did not set out to identify the specific visual and olfactory cues that underlie attraction behaviors in this experiment. Rather, we aimed to investigate the role of composite visual and olfactory cues in innate attraction. We find that at close range (1 m), prolonged exposure to visual cues alone from *L. camara* is sufficient for attraction while olfactory cues are dispensable. It is likely that *L. camara* presents a combination of visual cues that are innately attractive to monarchs. Monarchs have an innate preference for orange (primary) and yellow (secondary) color (Blackiston et al. 2011), which were prominent in the *L. camara* used in this experiment. Monarchs probably also show attraction to more general flowering plant features (green leaves, symmetric petals, etc.) as they have been shown to be attracted to visual features of other flowering plants, for example, milkweed (Garlick 2007). We cannot assert if visual responses observed in this experiment reflect monarch color preference, are behaviors specific to *L. camara* , or are more general plant preferences. Repeating these experiments with different nectar plants and specific artificial cues will clarify these questions.

Temporal differences in behavior reveal attraction‐ versus perception‐dependent behaviors. Sustained directed flight toward the plant might indirectly indicate the salience of attractive cues or motivation to pursue the plant, beyond simply the perception of attractive cues. Monarchs showed increased directional flight over time toward an uncovered plant placed 1 m away, but no difference over time when the plant was covered. These results indicate that there is a quantitative difference in the flight toward the plant when only visual cues are present versus both visual and olfactory cues and further indicate that flight toward the plant in this assay is an attraction response when the plant is perceived within a short (1 m) distance range. The timed difference in flight toward the uncovered plant may suggest that individuals not only perceived the plant at this distance, but they potentially grew more motivated to fly toward it over the course of the trial. This is more likely than the possibility that the first flight bin included a brief uncertainty or acclimation period since the visual‐only treatment did not show a similar difference in first versus second bin flight. Additional experiments are required to more rigorously test this idea.

When the plant was presented farther away (3 m), individuals did not show attraction to the plant in the first or second time bins. One interpretation of no attraction in the 3 m‐combination group is that butterflies were unable to perceive the plant at this distance. This aligns with the findings of Garlick (2007) in which monarchs were attracted to visual plant signals at 2 m, leading these authors to propose that monarch visual perception is short range. In contrast, however, olfactory perception extends tens of meters in the wild (Fisher and Bradbury 2021) and is suspected to extend even farther under certain weather conditions (e.g., wind speed, direction) (Grant et al. 2022). The lack of wind in our experiment may have reduced the salience of olfactory cues. Nevertheless, additional results are consistent with the interpretation of an inability to perceive the plant at 3 m. Individuals in the 3 m‐combination group spent a similar proportion of their trial flying directionally as did individuals in the 1 m groups (Figure 4a), but they did not choose to fly toward the plant. As well, individuals from the 3 m‐combination treatment spent less time circling than did individuals in the 1 m groups, reaching significance for the 1 m‐visual group (Figure 4c). “Circling” or “spiraling” is a behavior that is described in butterflies when individuals are in specific pursuit of objects, often other individuals (Imafuku and Ohtani 2006). We speculate that circling in this assay may be a potential escape response in which the butterflies detect a nearby attractive stimulus that they intend to reach and thus fly vigorously in circles in order to free themselves of the apparatus. An alternative idea to explain the failure of individuals to fly directionally toward the plant at 3 m is that monarchs perceived the plant yet did not pursue it, possibly due to low salience of the presented cues. Pollinators may alter their responses when sensory cues are in conflict or are uncoupled (Riffell 2020). For example, hawkmoths ( *Manduca sexta* ) have trouble tracking an artificial flower if visual and mechanosensory cues are mismatched (Roth et al. 2016). Such behavior might reflect an efficient search strategy in which only resources that are recognized with high certainty within a specific distance elicit energy‐intensive directional flight. Monarchs are efficient foragers in grass‐covered landscapes (Fisher and Bradbury 2022). They explore nectar resources in tortuous paths in high‐density resource environments (Zalucki and Kitching 1982; Zalucki 1983), suggesting an ability to respond to cue salience at short distances. The most frequent flight steps that monarchs took in a varied landscape prairie were < 5 m and were associated with foraging (Fisher et al. 2020). Our study suggests that visual cues are most relevant for guiding these flight movements at this spatial scale. Monarchs may use long‐term memory to refine their foraging search during periods of localized feeding (Konnerth et al. 2023).

The combination of multiple cue modalities enhances attraction to flowers in many insect species (Raguso and Willis 2005; Chow and Frye 2008). While we find that visual cues are primary for nectar plant attraction, olfactory cues impact the attractiveness of visual cues from *L. camara* . Whereas combined visual and olfactory cues elicited a sustained attraction response that grew stronger over time, attraction was unchanged between the first and second flight halves in the visual‐only treatment. This indicates that monarchs could perceive the plant using visual cues, but their motivation to pursue the plant did not grow over time when olfactory cues were missing. Olfactory cues may increase the salience of a visual cue, influence the valence timescale of a visual cue (i.e., increase the time by which an attractive cue is considered attractive with no reward or reinforcement), or specifically enhance flight tendency (i.e., visual cues are used for perception and olfactory cues are used to stimulate flight). Details of the role of olfactory cues in monarch foraging will benefit from experiments that present visual cues with olfactory cues in more specific ways [such as wind‐directed cues in a Y‐maze olfactometer (Zalucki and Kitching 1982) or wind tunnel (Zalucki and Kitching 1984)].

In the generalist hoverfly model, multimodal cues are required to effectively identify a broad array of nutritive resources (Mishra et al. 2024) across different environments (Nordström et al. 2017). We find that visual cues are sufficient for monarchs to be attracted to *L. camara* , but attraction is enhanced by olfactory cues. An important difference in our experiment is that individuals chose between putatively attractive cues versus no cues at all. Therefore, our experiment does not address how cues are used to identify specific nectar resources among a range of choices. It is possible that monarchs utilize additional modalities differently when resources must be distinguished among. When milkweed plants are presented in a grass‐covered landscape, monarchs show innate attraction to visual and olfactory cues when presented separately, and they act synergistically when paired (Kelber 1997). This attraction may reflect context‐dependent cue salience, or it may reflect a difference in cue use for identifying nectar resources versus oviposition sites. In contrast to their generalist nectaring strategy, monarchs exclusively oviposit onto species of Asclepiacae (genera *Asclepias*, *Gomphocarpus*) (Grant et al. 2022).

Intrapopulation variation in innate sensory attraction is prevalent and can have significant consequences for species. Female pipevine swallowtails ( *B. philenor* ) show a bimodal distribution for leaf shape preference (Fisher et al. 2021). Variation in innate preference for violet color is correlated with differences in nectar foraging rate in bumblebees ( *B. terrestris* ; Raine and Chittka 2007), indicating the adaptive potential of innate sensory perception. We find large variation for innate nectar plant attraction among individuals within groups in our experiments (Figures 2 and 4). It is not yet known, but is of interest, if this behavioral variation in monarchs is determined genetically, or what specific fitness benefit this innate attraction may provide. Moreover, innate sensory attraction often shows sexual dimorphism, reflecting ecologically driven sex‐specific priorities. Males and female swallowtail butterflies (*Papilio demoleus*) have innate preferences for different cues (visual and olfactory cues, respectively) during foraging and courtship (Grant et al. 2022). Females, but not males, shift color preference when presented a specific odor cue in the swallowtail *Papilio xuthus* (Kelber 2002). Despite these trends, we do not find sex‐specific perception differences (Table 2). This is consistent with the finding that although male and female monarchs utilize a common resource landscape differently, they do not differ in their overall straight‐line headings and show no overall directionality (Zalucki and Kitching 1982), which is more akin to what is measured in our assay. Monarchs also have sexually monomorphic eye pigmentation (Kadow 2019), consistent with our finding that neither sex has stronger visual acuity as detected in this assay.

## 5 Conclusion

We show that generalist foraging monarch butterflies have evolved an innate attraction to visual cues from specific nectar resources ( *L. camara* ) that control their behavior prior to learning about its reward status. Our relatively straightforward and inexpensive setup can capture different features of sensory perception in these butterflies, including distinguishing contributions of different sensory modalities and giving initial insight into the salience of these cues. This accessible assay may be useful for other researchers in a range of systems as an initial tool to study sensory perception.

## Author Contributions

**Darene A. E. Assadia:** conceptualization (equal), data curation (lead), formal analysis (lead), funding acquisition (supporting), investigation (equal), methodology (lead), validation (equal), visualization (lead), writing – original draft (equal), writing – review and editing (equal). **Delbert A. Green II:** conceptualization (equal), data curation (supporting), formal analysis (supporting), funding acquisition (lead), investigation (equal), methodology (supporting), project administration (lead), resources (lead), supervision (lead), validation (equal), visualization (supporting), writing – original draft (equal), writing – review and editing (equal).

## Conflicts of Interest

The authors declare no conflicts of interest.

## Supporting information


Data S1.

## Data Availability

All raw data values required to repeat analyses presented in this paper are included in the Supporting Information S1 associated with this manuscript.
